# Supplementary material for: Metal and Phosphate Ions Show Remarkable Influence on the Biomass Production and Lipid Accumulation in Oleaginous Mucor circinelloides
Source: J Fungi (Basel). 2020 Oct 30;6(4):260. doi: 10.3390/jof6040260 (PMC7711463; doi:10.3390/jof6040260)
Supplement: Supplementary file 1 [file jof-06-00260-s001.pdf]

Supplementary Materials

Metal and phosphate ions show remarkable influence on the biomass production and lipid accumulation in oleaginous Mucor circinelloides

Table S1: Concentrations of salts used for regulating metal ions and inorganic phosphorus levels in the growth media

	Condition label and salt concentration (g/L)									
Salts	0.01	0.10	0.25	0.5	1 (R)	2	4	10	100	1000
$MgSO_4.7H_2O$	0.015	0.15	-	-	1.5	-	-	15	150	1500
$CaCl_2.2H_2O$	0.001	0.01	-	-	0.1	-	-	1	10	100
$FeCl_3·6H_2O$	-	-	-	-	0.008	-	-	0.08	0.8	8
$ZnSO_4·7H_2O$	-	-	-	-	0.001	-	-	0.01	0.1	1
$CoSO_4·7H_2O$	-	-	-	-	0.0001	-	-	0.001	0.01	0.1
$CuSO_4·5H_2O$	-	-	-	-	0.0001	-	-	0.001	0.01	0.1
$MnSO_4·5H_2O$	-	-	-	-	0.0001	0.0002	-	0.001	0.01	0.1
KH_2PO_4	-	-	1.75	3.5	7	14	28	-	-	-
Na_2HPO_4	-	-	0.5	1	2	4	8	-	-	-

Table S2: pH of culture supernatant

Pi	0.25	0.5	1	2	4
R	2.17	2.33	2.88	4.81	5.67
0Ca	2.23	2.35	2.86	4.33	5.72
0.01Ca	2.25	2.41	2.94	4.60	5.54
0.1Ca	2.23	2.37	2.93	4.62	5.56
10Ca	2.17	2.31	2.83	4.25	5.30
10Ca0Mg	4.01	4.76	5.54	5.89	5.96
0.01Mg	2.30	2.56	3.15	4.63	5.73
0.1Mg	2.17	2.39	2.92	4.27	5.59
0Co	2.17	2.33	2.89	4.61	5.68
10Co	2.24	2.35	2.86	4.24	5.71
100Co	2.25	2.39	2.87	4.04	5.57
1000Co	2.22	2.37	2.81	3.85	5.38
0Cu	2.19	2.30	2.76	4.20	5.75
10Cu	2.18	2.33	2.94	4.75	5.67
100Cu	2.20	2.34	2.95	4.50	5.60
1000Cu	2.14	2.32	2.93	4.30	5.54
0Fe	2.09	2.23	2.82	3.97	5.56
10Fe	2.10	2.26	2.77	4.05	5.45
100Fe	2.08	2.22	2.65	3.50	5.26
1000Fe	1.62	1.62	1.85	2.35	2.79
10Ca0Mg	4.01	4.76	5.54	5.89	5.96
10Mn	2.20	2.33	2.86	3.97	5.67
100Mn	2.11	2.28	2.88	4.19	5.56
1000Mn	2.11	2.26	2.80	3.89	5.60
0Zn	2.99	3.49	4.19	5.70	5.81
10Zn	2.12	2.28	2.85	4.37	5.67
100Zn	2.07	2.25	2.85	4.05	5.70
1000Zn	2.03	2.25	2.86	3.78	4.97

Table S3: Biomass concentration (g/L)

Pi	0.25	0.5	1	2	4
R	1.81	3.05	9.80	10.89	11.10
0Ca	2.47	4.70	12.37	9.73	10.24
0.01Ca	1.70	3.07	9.49	9.87	9.34
0.1Ca	1.73	3.44	9.91	10.80	11.37
1Ca	1.81	3.05	9.80	10.89	11.10
10Ca	1.87	3.29	10.86	12.16	9.80
10Ca0Mg	0.00	0.00	0.00	0.00	0.00
0.01Mg	0.51	1.24	1.50	4.47	4.36
0.1Mg	0.94	2.60	8.39	10.36	11.10
1Mg	1.81	3.05	9.80	10.89	11.10
0Co	1.59	3.14	10.34	12.33	12.31
1Co	1.81	3.05	9.80	10.89	11.10
10Co	1.26	2.51	10.44	12.47	13.23
100Co	1.27	3.29	10.89	11.97	12.41
1000Co	0.90	2.11	7.84	10.13	12.06
0Cu	1.17	2.71	8.84	10.33	11.79
1Cu	1.81	3.05	9.80	10.89	11.10
10Cu	1.83	3.61	11.07	12.37	12.74
100Cu	1.56	3.37	9.97	11.94	12.51
1000Cu	2.30	3.20	9.89	10.73	10.71
0Zn	0.57	0.73	1.27	0.90	0.57
1Zn	1.81	3.05	9.80	10.89	11.10
10Zn	1.86	3.30	10.56	11.06	12.70
100Zn	2.54	4.09	12.90	10.70	12.81
1000Zn	2.79	4.44	12.21	9.80	8.36
0Fe	2.30	3.43	5.69	7.54	9.31
1Fe	1.81	3.05	9.80	10.89	11.10
10Fe	1.74	3.50	10.81	10.97	11.24
100Fe	0.00	2.61	8.54	10.77	11.84
1000Fe	0.00	0.00	0.00	2.34	7.89
0Mn	1.76	3.61	9.24	10.73	10.73
1Mn	1.81	3.05	9.80	10.89	11.10
2Mn	1.49	2.50	10.13	10.24	12.60
3Mn	1.66	1.60	8.26	7.71	11.27
10Mn	1.57	2.73	9.27	10.30	12.60
100Mn	1.47	2.90	10.49	10.47	11.50
1000Mn	1.71	3.13	10.13	9.51	12.93

Table S4: Fatty acid profiles (%)

Sample	C14:0	C16:0	C16:1	C18:0	C18:1n9c	C18:2n6c	C18:3n6	others
R Pi1	1.51	21.23	1.80	4.68	38.07	14.70	13.99	4.02
R Pi2	1.86	17.35	4.97	3.49	42.91	12.74	11.44	5.24
R Pi4	1.90	16.82	6.00	3.08	43.87	12.45	10.60	5.30
0Ca Pi1	1.39	24.51	1.46	4.72	37.17	17.69	9.44	3.62
0Ca Pi2	1.79	18.75	4.46	3.13	40.84	14.09	9.26	7.67
0Ca Pi4	1.76	19.29	5.20	3.00	42.39	13.97	10.55	3.85
0.01Ca Pi1	1.85	24.12	0.03	5.21	37.33	15.58	12.28	3.61
0.01Ca Pi2	1.86	17.89	4.54	3.45	41.83	13.51	11.17	5.73
0.01Ca Pi4	2.12	16.86	5.62	4.20	41.55	13.25	10.28	6.13
0.1Ca Pi1	1.32	22.86	1.45	4.85	36.60	15.12	13.05	4.74
0.1Ca Pi2	1.79	18.63	4.59	3.27	41.74	14.01	11.95	4.02
0.1Ca Pi4	1.88	18.56	5.36	3.09	41.82	13.70	10.68	4.90
10Ca Pi1	1.55	23.02	1.73	4.68	36.25	14.92	13.76	4.08
10Ca Pi2	1.91	16.53	5.22	3.31	41.41	13.54	11.92	6.16
10Ca Pi4	1.84	13.46	6.65	2.41	43.80	14.81	11.70	5.34
0.01Mg Pi1	5.83	33.89	0.12	5.88	0.46	7.27	28.30	18.26
0.01Mg Pi2	2.34	24.42	1.24	5.13	27.93	7.04	20.27	11.64
0.01Mg Pi4	2.32	22.17	3.26	3.02	31.36	8.18	19.28	10.41
0.1Mg Pi1	0.04	23.86	1.26	4.26	33.39	16.45	15.67	5.06
0.1Mg Pi2	0.05	18.36	4.00	3.82	40.24	14.30	13.45	5.77
0.1Mg Pi4	0.05	16.29	6.03	3.15	41.81	14.72	11.76	6.20
0Co Pi1	1.35	22.64	1.48	4.27	35.28	16.22	14.99	3.77
0Co Pi2	1.78	18.32	4.63	3.25	41.47	13.95	12.44	4.17
0Co Pi4	1.92	17.34	6.01	2.75	42.37	13.68	11.42	4.51
10Co Pi1	1.43	22.70	1.49	4.38	35.45	16.94	14.53	3.08
10Co Pi2	2.04	20.34	4.26	4.25	40.76	13.44	11.24	3.68
10Co Pi4	2.21	21.26	0.02	3.82	44.00	13.95	11.19	3.56
100Co Pi1	1.47	22.76	1.34	5.04	34.84	17.05	14.39	3.11
100Co Pi2	2.09	19.52	4.12	4.70	40.67	12.90	10.78	5.22
100Co Pi4	2.39	19.59	5.47	4.32	41.11	13.08	10.48	3.57
1000Co Pi1	1.79	22.97	1.14	8.63	30.24	16.85	12.34	6.06
1000Co Pi2	2.26	20.71	2.53	9.00	34.01	13.07	10.85	7.58
1000Co Pi4	2.49	22.66	3.42	10.56	34.29	11.53	9.03	6.02
0Cu Pi1	1.50	22.73	1.61	3.64	35.60	15.65	15.85	3.41
0Cu Pi2	1.72	18.45	4.38	3.24	40.87	13.50	12.98	4.85
0Cu Pi4	1.88	17.59	5.77	2.56	41.17	13.50	11.59	5.93
10Cu Pi1	1.36	22.43	1.49	4.27	35.53	15.84	14.56	4.52
10Cu Pi2	1.69	18.15	4.70	3.18	42.27	13.26	12.19	4.56
10Cu Pi4	1.78	17.31	5.86	2.56	42.84	13.51	11.39	4.76
100Cu Pi1	1.39	22.15	1.77	4.37	36.06	15.75	13.72	4.78
100Cu Pi2	1.90	18.18	4.93	3.29	41.19	13.42	11.53	5.56
100Cu Pi4	1.90	16.99	6.44	2.67	42.90	13.36	10.53	5.22
1000Cu Pi1	1.50	22.23	2.09	4.47	36.75	15.17	13.19	4.61
1000Cu Pi2	1.84	16.94	5.45	3.00	42.20	13.71	10.49	6.37
1000Cu Pi4	1.80	14.52	7.20	2.06	43.68	13.69	9.64	7.40

Sample	C14:0	C16:0	C16:1	C18:0	C18:1n9c	C18:2n6c	C18:3n6	others
0Fe Pi1	1.98	21.77	1.60	6.20	32.80	17.98	10.27	7.40
0Fe Pi2	1.82	18.01	3.20	4.50	39.79	12.65	12.75	7.27
0Fe Pi4	2.09	16.86	6.78	0.00	44.02	13.22	11.24	5.79
10Fe Pi1	0.04	24.21	0.02	4.78	36.40	15.94	14.48	4.12
10Fe Pi2	0.05	16.33	5.32	3.63	42.27	13.90	11.39	7.10
10Fe Pi4	0.05	15.44	7.10	4.24	41.37	13.34	10.68	7.79
100Fe Pi1	0.06	23.33	0.02	3.59	35.60	15.11	17.29	4.99
100Fe Pi2	0.06	20.13	3.80	3.81	40.83	13.76	12.67	4.94
100Fe Pi4	2.18	16.08	6.45	3.39	41.35	12.46	10.27	7.81
0Mn Pi1	1.41	22.15	1.66	4.84	37.52	13.97	14.74	3.71
0Mn Pi2	1.64	17.55	4.71	2.96	41.40	12.60	12.12	7.00
0Mn Pi4	2.01	17.58	6.35	2.85	42.22	12.68	11.07	5.24
10Mn Pi1	1.66	22.34	1.69	5.14	35.63	15.50	14.12	3.93
10Mn Pi2	1.79	17.10	4.34	3.20	41.52	14.16	12.83	5.05
10Mn Pi4	1.86	18.29	5.79	2.87	42.05	13.51	11.30	4.34
100Mn Pi1	0.04	22.80	1.39	3.97	34.53	16.73	15.90	4.63
100Mn Pi2	1.80	16.79	4.36	3.48	41.88	13.84	11.95	5.90
100Mn Pi4	1.93	17.08	5.95	2.69	42.43	13.77	11.21	4.94
1000Mn Pi1	0.06	22.55	1.53	3.92	34.40	16.52	16.25	4.77
1000Mn Pi2	0.05	16.67	4.54	3.47	40.81	14.53	13.12	6.81
1000Mn Pi4	0.05	18.37	6.08	2.86	42.68	14.04	11.34	4.59
10Zn Pi1	1.68	21.81	1.68	5.98	36.69	15.52	12.44	4.19
10Zn Pi2	1.87	19.15	4.11	3.57	41.41	13.56	11.41	4.91
10Zn Pi4	1.78	19.36	4.61	3.38	42.13	13.14	11.03	4.58
100Zn Pi1	1.43	23.30	1.24	5.54	35.65	15.78	13.61	3.44
100Zn Pi2	2.01	18.81	4.07	4.04	40.54	13.74	10.37	6.43
100Zn Pi4	1.88	20.14	4.27	3.66	42.25	13.28	11.10	3.42
1000Zn Pi1	1.69	23.70	1.05	8.28	34.89	14.64	11.77	3.98
1000Zn Pi2	2.39	22.11	2.30	8.90	35.68	13.47	8.60	6.54
1000Zn Pi4	2.03	19.34	4.41	3.77	41.08	12.82	9.45	7.09

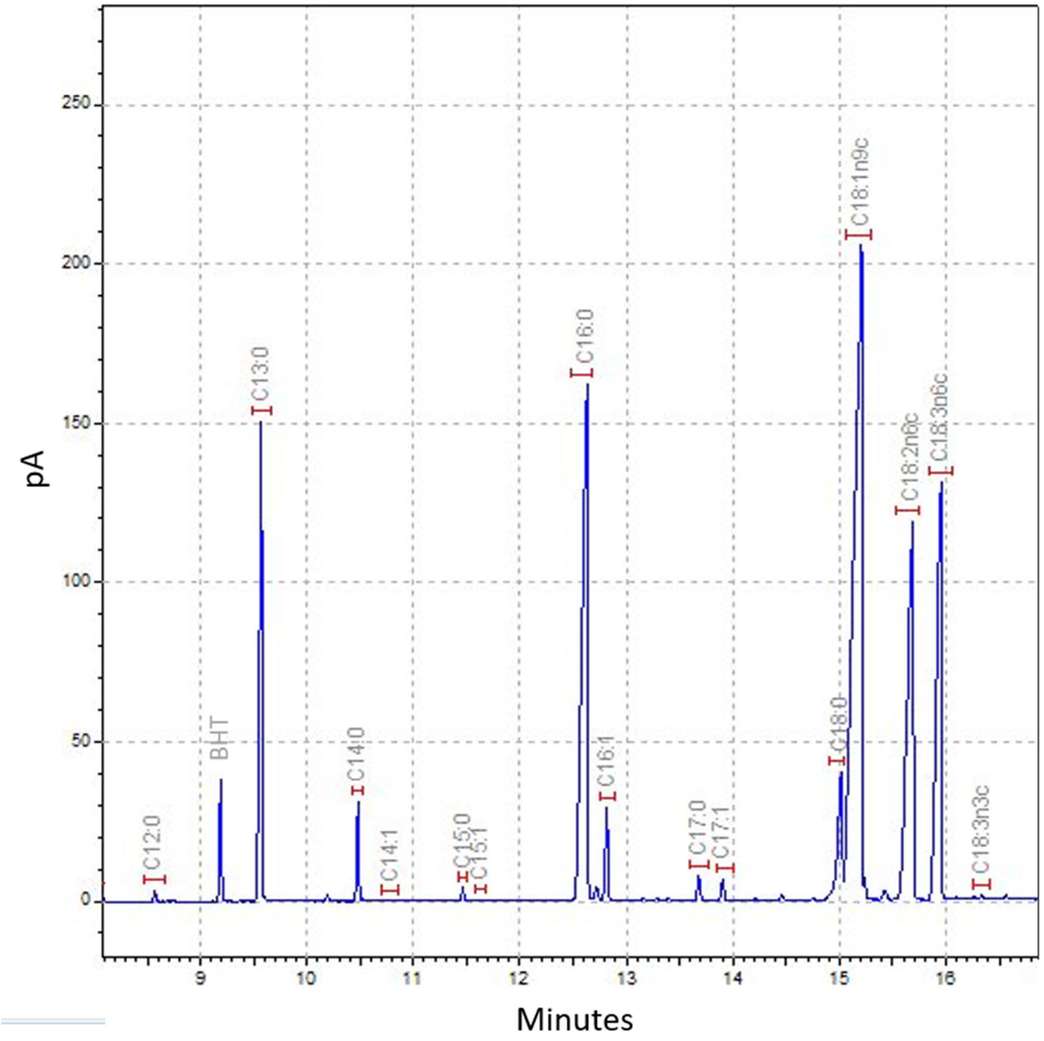

Figure S1: Example chromatogram, *Mucor circinelloides* grown in Pi1-R condition.

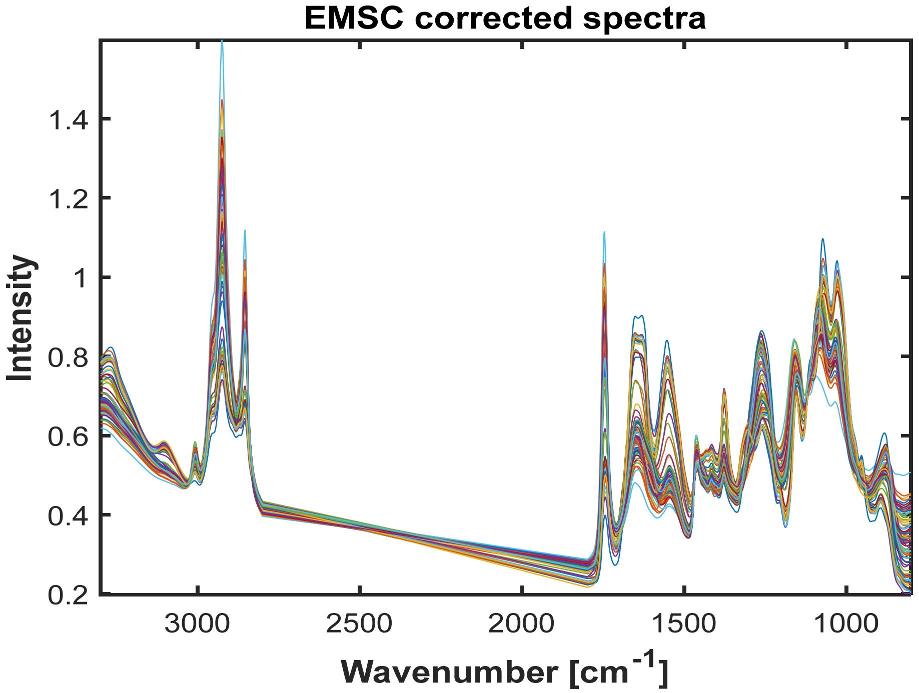

Figure S2: EMSC corrected FTIR-HTS spectra of *Mucor circinelloides* biomass

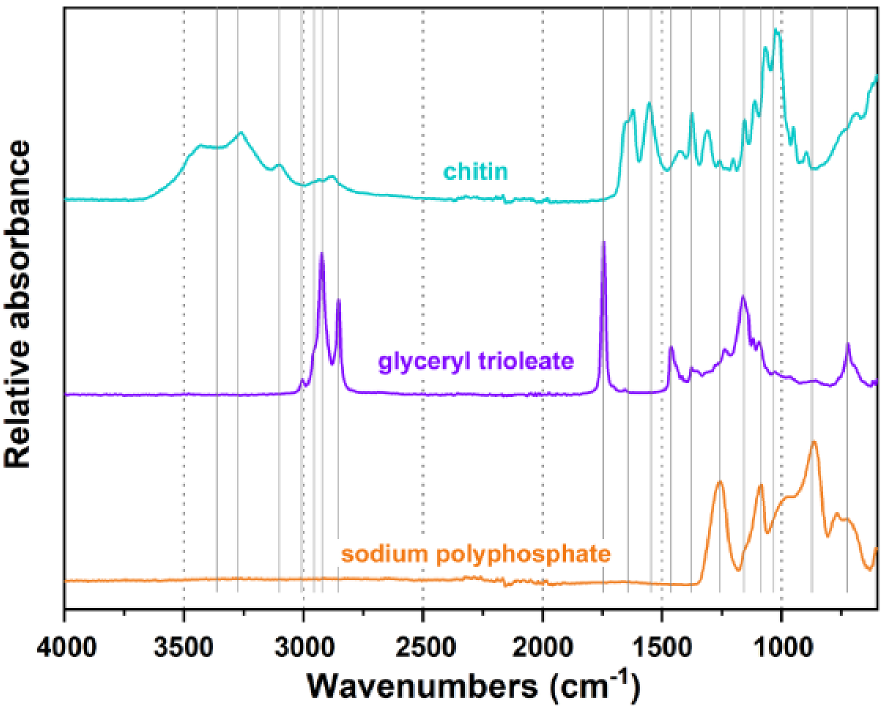

Figure S3: FTIR spectra of reference materials. Adapted from Dzurendova et al. [1]

[1] Dzurendova, S.; Zimmermann, B.; Kohler, A.; Tafintseva, V.; Slany, O.; Certik, M.; Shapaval, V. Microcultivation and FTIR spectroscopy-based screening revealed a nutrient-induced co-production of high-value metabolites in oleaginous Mucoromycota fungi. *PloS one* 2020, *15*, e0234870

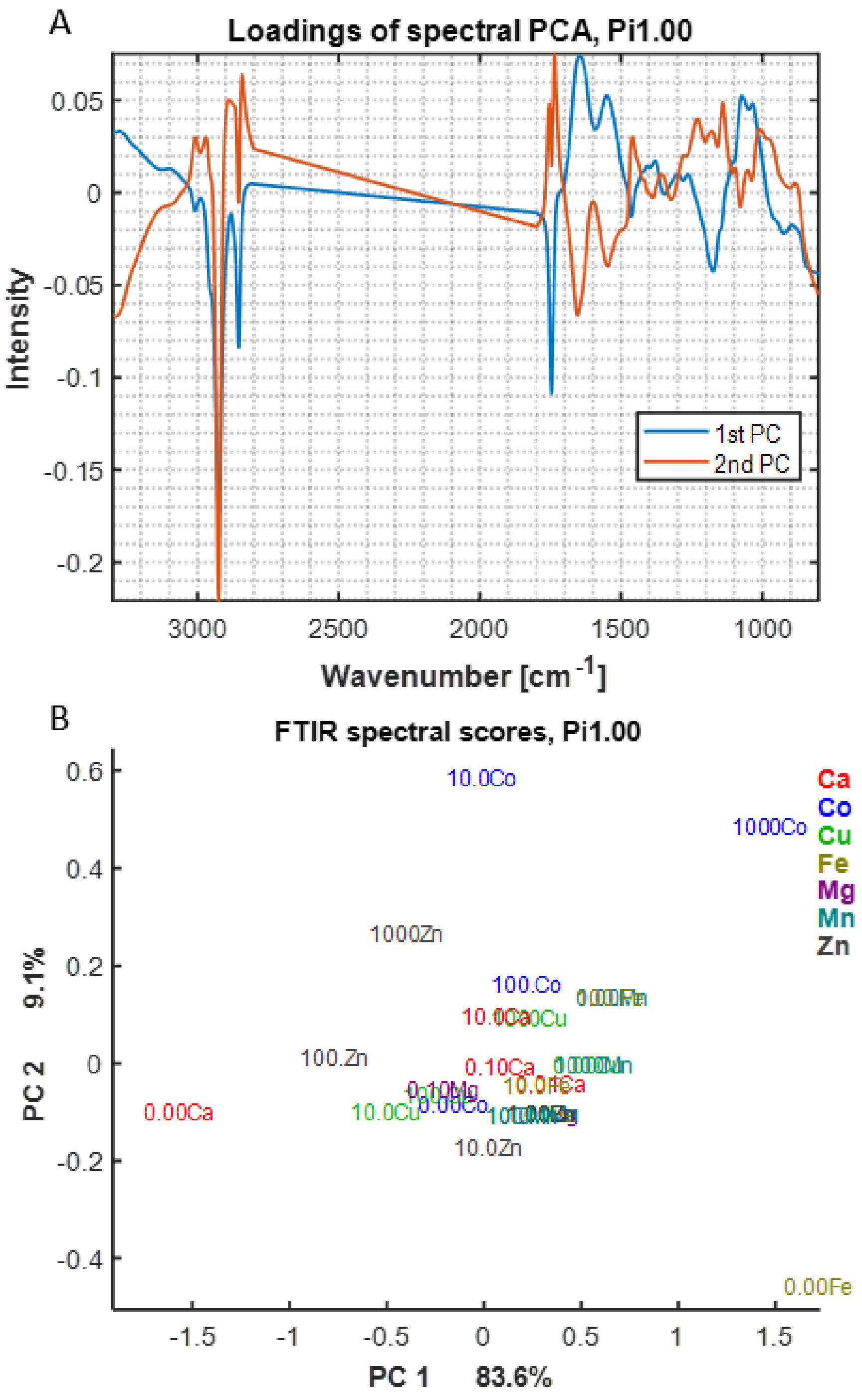

Figure S4: PCA analysis of FTIR-HTS spectra of *Mucor circinelloides* biomass grown under Pi1 level. The loadings of spectral PCA (A), the score plot (B).

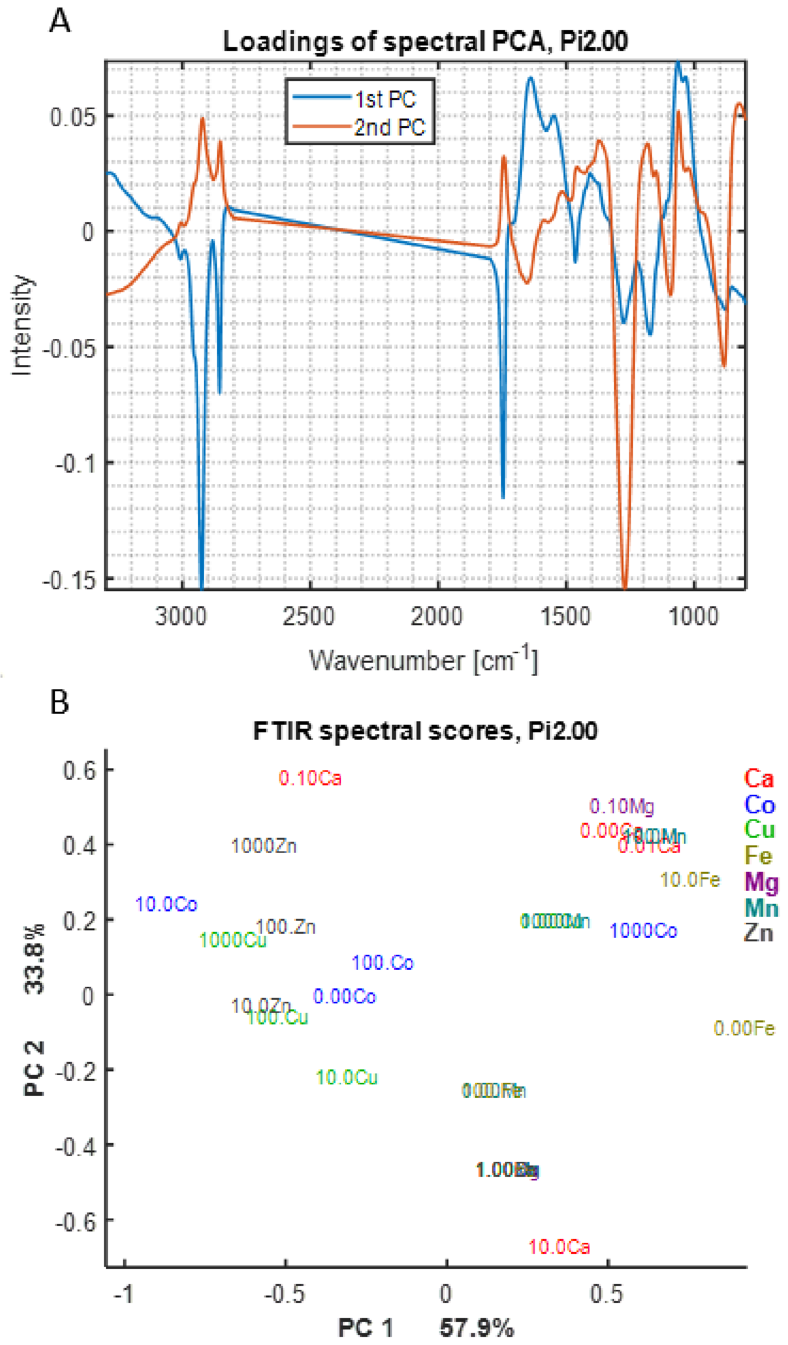

Figure S5: PCA analysis of FTIR-HTS spectra of *Mucor circinelloides* biomass grown under Pi2 level. The loadings of spectral PCA (A), the score plot (B).

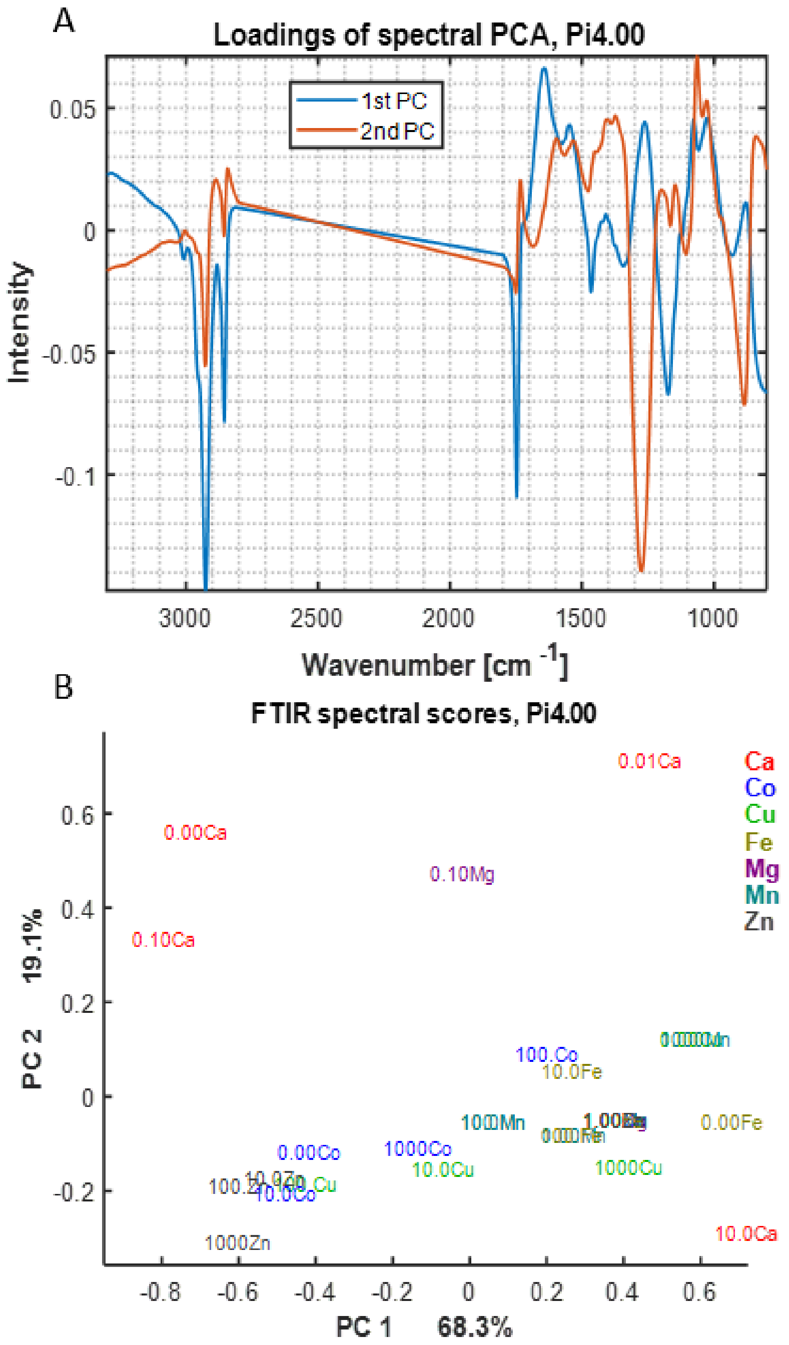

Figure S6: PCA analysis of FTIR-HTS spectra of *Mucor circinelloides* biomass grown under Pi4 level. The loadings of spectral PCA (A), the score plot (B).
